# Muscle power amplification may enhance sprint performance and broaden the thermal performance of the lizard *Sceloporus occidentalis*. II: Muscle power and series elastic element properties

**DOI:** 10.1242/jeb.252629

**Published:** 2026-10-23

**Authors:** Anthony J. Cobos, Jordan Balaban, Emanuel Azizi, Natalie C. Holt

**Affiliations:** ^1^ Department of Evolution, Ecology, and Organismal Biology (EEOB), University of California, Riverside, 900 University Ave, Riverside, CA 92521, USA; ^2^ Biology Department, Barnard College, Columbia University, 3009 Broadway, New York, NY 10027, USA; ^3^ Department of Ecology and Evolutionary Biology, Charlie Dunlop School of Biological Sciences, University of California, Irvine, Irvine, CA 92697, USA

**Keywords:** Elastic strain energy, Muscle–tendon unit, Temperature, Fence lizard

## Abstract

Skeletal muscle is reliant on the myosin ATPase enzyme to power locomotion, making muscle and locomotor performance thermally sensitive. However, muscles performance is often enhanced by elastic tendons, and the power amplification they enable is thought to increase sprint performance and its thermal robustness. The previous study suggests *in vivo* ankle muscle powers that exceed predicted muscle powers and may contribute to the high thermal robustness of sprint performance in *S. occidentalis*. Here, we directly measure gastrocnemius muscle power and its thermal sensitivity, compare this to *in vivo* ankle muscle power to quantify power amplification, and determine the potential for the gastrocnemius muscle–tendon unit (MTU) to store the required elastic strain energy. Peak gastrocnemius muscle power was ∼83, 156 and 371 W kg^−1^ at 15, 25 and 35°C with *Q*_10_ values of ∼1.9–2.4. Peak *in vivo* muscle power at the ankle was ∼180, 622 and 881 W kg^−1^, indicating a ∼2.2-, 4.1- and 2.4-fold power amplification, at 15, 25 and 35°C, respectively. The fixed-end compliance of the gastrocnemius was ∼17%, series elastic elements experienced an ∼12% strain, and the muscle fibers stored ∼17 J kg^−1^. Hence, this species clearly uses power amplification in sprinting at all temperatures, and the high degree of power amplification at 25°C probably increases thermal robustness of sprint performance at 25–35°C. The degree of elastic strain energy storage observed in this MTU is comparable with avian, mammalian and anuran MTUs known to store and utilize elastic strain energy and modestly amplify muscle power.

## Teaser

**Summary:** Thermal sensitivity of the gastrocnemius muscle of *Sceloporus occidentalis* and the capacity for elastic strain energy storage in this muscle–tendon unit indicate that power amplification contributes to sprint performance and thermal robustness.

## INTRODUCTION

There is an inextricable link between an animal's morphological, physiological and biochemical traits and its behavior, fitness and ecology (Arnold, 1983; Garland and Losos, 1994). Skeletal muscle is the biological motor and thus, an understanding of muscle performance across ecologies is critical to understanding behavioral capacity and fitness. However, muscle does not function in isolation: it acts on the environment via rigid skeletal elements (Biewener, 1990) and, often, elastic tendons (Alexander and Bennet-Clark, 1977; Pollock and Shadwick, 1994). Hence, locomotion is an emergent property resulting from the interaction of these elements of the musculoskeletal system and their environment (Dickinson et al., 2000), and an understanding of the role of underlying traits necessitates study across multiple spatial scales in physiologically and ecologically relevant contexts (Marsh and Bennett, 1986a,b; Marsh and Olson, 1994; Daley et al., 2009).

Skeletal muscle converts chemical energy into the mechanical output required for the majority of animal movements. Upon muscle activation, the contractile proteins actin and myosin use ATP hydrolysis by the myosin ATPase enzyme to drive cyclical force- and work-generating crossbridge interactions between them. The kinetics of crossbridge cycling give rise to the maximum muscle shortening speed (*V*_max_) and the inverse relationship between muscle force and velocity that constrain muscle performance (Hill, 1938; Piazzesi et al., 2003; Mendoza et al., 2023b). Elastic tendons, and other series elastic elements (SEEs) such as aponeuroses (Alexander and Bennet-Clark, 1977; Pollock and Shadwick, 1994; Zuurbier et al., 1994; Azizi and Roberts, 2009; Herzog, 2019; Holt, 2020), can circumvent the constraints of *V*_max_ and the force–velocity relationship. SEEs are passive structures and so cannot convert chemical energy into work. However, they can store elastic strain energy during stretch and return it at a later time during recoil with relatively low hysteresis (Wilson and Goodship, 1994), so enabling a temporal decoupling of the work done by muscles from that done on the environment. This can increase the fraction of a muscle's work capacity that can be delivered by amplifying muscle power (Peplowski and Marsh, 1997; Roberts and Marsh, 2003; Roberts and Azizi, 2011) or increasing muscle kinetic energy capacity (Labonte and Holt, 2024). In this phenomenon, muscle does work slowly on SEE prior to a ballistic movement, so storing elastic strain energy and avoiding limits to muscle work imposed by muscle shortening velocity. This energy is then returned rapidly during recoil enabling more work to be done more rapidly, and therefore with a higher power, than would be possible with muscle alone, at least in small species (Sutton et al., 2019; Labonte and Holt, 2024). Power amplification can therefore enhance organismal performance above that possible using muscle alone. However, it is thought that power amplification can not only circumvent these intrinsic constraints arising from crossbridge cycling, but also the extrinsic constraints of the environment such as temperature (Anderson and Deban, 2010; Deban and Lappin, 2011; Deban and Scales, 2016).

Temperature is one of the most significant ecological factors impacting organismal performance, particularly in ectotherms. It acts as a selective pressure, shaping animal phenotypes and distributions (Angilletta, 2009), owing to its effect on enzyme kinetics (Peterson et al., 2007). In muscle, temperature affects the kinetics of enzymatic processes including that of the myosin ATPase, and so the rate of crossbridge cycling. Hence, muscle performance is temperature sensitive (Marsh and Bennett, 1986a,b; Bennett, 1990; Garland, 1994; Angilletta, 2009; James, 2013). Static measures of muscle performance, such as isometric force, have low *Q*_10_ (change in performance with 10°C temperature increase) values of ∼1.1 (Ranatunga, 1982, 1998; Rall and Woledge, 1990; Coupland et al., 2001; James, 2013). However, more dynamic measures such as *V*_max_ have a greater dependence on temperature with *Q*_10_ values of 2–3 owing to their greater dependence on rates of crossbridge cycling (Hill, 1938; Johnston and Gleeson, 1984; Marsh and Bennett, 1986a,b; Coughlin et al., 1996). Muscle power, the product of force and velocity, is the most temperature sensitive metric of muscle performance with *Q*_10_ values often >3 (Bennett, 1984; Hirano and Rome, 1984; Marsh and Bennett, 1986a; Rall and Woledge, 1990; Swoap et al., 1993; Ranatunga, 1998; James, 2013; Olberding and Deban, 2017). Whereas muscle performance, particularly *V*_max_ and power, is highly temperature sensitive, the passive nature of SEEs means that their performance is largely temperature independent (Rigby et al., 1959; Kubo et al., 2005).

This temperature insensitivity of SEEs, and low temperature sensitivity of more static aspects of muscle performance, may explain why the temperature sensitivity of muscle does not always translate to the temperature sensitivity of organismal performance. In power-amplified systems, muscle can still do substantial work slowly at low temperature and elastic tissues are still able to recoil rapidly (Bennett, 1984; James, 2013), so allowing for high take-off powers despite low muscle power. Ballistic movements like tongue projection in chameleons and salamanders (Anderson and Deban, 2010; Deban and Richardson, 2011) and frog jumping (Peplowski and Marsh, 1997; Astley and Roberts 2014; Mendoza and Azizi, 2021), have been shown to use power amplification to somewhat circumvent thermal constraints.

A discrepancy between muscle and organismal thermal sensitivity has been demonstrated in the western fence lizard where the temperature sensitivity of burst sprint speed is much lower than that of the iliofibularis muscle (Marsh and Bennett, 1986a,b). Our previous study in this series expanded on this early finding and used inverse dynamics to predict maximal apparent instantaneous *in vivo* muscle powers at joints during acceleration at 15, 25 and 35°C. The high predicted joint powers, particularly at the ankle, in this study suggest power amplification at this joint during the first, ballistic, acceleration step (Balaban et al., 2026). However, empirical data on the power output of the major muscle responsible for ankle extension, the gastrocnemius (Reilly and Delancey, 1997; Higham et al., 2011) and its temperature sensitivity are not currently available, and the potential for elastic strain energy in the SEEs associated with the gastrocnemius has not been determined. Tendons and aponeuroses vary widely in their capacity for elastic strain energy storage (Alexander and Bennet-Clark, 1977; Pollock and Shadwick, 1994; Ettema, 1996; Holt and Mayfield, 2023). And the ability of small running and hopping species to store energy in SEEs during cyclical locomotion has been questioned and remains unclear (Biewener and Blickhan, 1988; Pollock and Shadwick, 1994; Biewener and Roberts, 2000; Biewener, 2005; Bullimore and Burn, 2005; Christensen et al., 2022). Hence, here, we determine the effect of temperature on the force–velocity relationship and maximum power output of the western fence lizard gastrocnemius muscle, and the capacity of this muscle–tendon unit to store the elastic strain energy required for power amplification in its series elastic elements.

## MATERIALS AND METHODS

### Study animals

Western fence lizards (*Sceloporus occidentalis* Baird and Girard 1852) were captured with approved California Department of Fish and Wildlife collecting permits (SCP-21014) in the areas surrounding University of California, Riverside and housed in UC Riverside's vivarium following approved animal care protocols (IACUC protocol 20210009). Lizards were housed individually in tanks measuring ∼50×30×30 cm and provided with a paper towel substrate, heat pads under one area of the tank and UV lights on a 12 h:12 h light:dark cycle. The room was held at a constant temperature of ∼24°C and had the same light cycle as the UV lights. *In vitro* experiments were conducted on the gastrocnemius and associated distal tendon of adult western fence lizards. Data on the thermal sensitivity of muscle force–velocity properties and power were generated from *n*=27 lizards, and data on the potential for elastic strain energy storage in the tendon were gathered from a further *n*=5 lizards. All lizards were deemed to be adults with a body mass range of ∼9–16 g.

### *In vitro* muscle preparation

Lizards were euthanized via an overdose of 5% inhaled isoflurane, followed by a double pithing protocol [following approved American Veterinary Medical Association (AVMA) guidelines; Leary et al., 2020]. The gastrocnemius muscle and its distal tendons were exposed and the gastrocnemius muscle–tendon unit (MTU) isolated from the limb by cutting out a small chunk of bone connected to the origin of the muscle, tying onto the end of the distal tendon just proximal to the calcaneus with inextensible Kevlar thread, and then cutting the calcaneus. To assess the contractile properties of this muscle and elastic properties of the tendon, the MTU was attached by its insertion to a dual-mode servomotor (305C-LR Dual-Mode Lever System, Aurora Scientific, Aurora, ON, Canada) using the Kevlar thread, and the proximal bone fixed in a stationary clamp (Balaban and Azizi., 2017; Holt and Azizi, 2014). During experiments, muscles were bathed in an oxygenated Ringer's solution (112 mmol l^−1^ NaCl, 3.3 mmol l^−1^ KCl, 2.5 mmol l^−1^ CaCl_2_, 1 mmol l^−1^ MgCl_2_, 20 mmol l^−1^ HEPES, 5 mmol l^−1^ dextrose; Jayasinghe and Launikonis, 2013) adjusted to pH 7.4 with NaOH. A flask of this Ringer's solution was maintained in a water bath, the temperature of which could be adjusted for each temperature condition and circulated to the muscle chamber (Mendoza et al., 2023a).

All recording and data processing were performed using data acquisition software (Igor Pro 8, WaveMetrics, Lake Oswego, OR, USA). Stimulation protocols were constructed (Igor Pro 8) (Holt and Azizi, 2014; Castro et al., 2022) and sent to the muscle using a stimulator (701C high-powered bi-phase stimulator, Aurora Scientific) and a DAQ AD board (CompacDAQ, National Instruments, Austin, TX, USA), and muscle force and length data were logged at a sampling frequency of 10,000 Hz using the same DAQ AD board (CompacDAQ, National Instruments). Supramaximal square wave pulses (amplitude 6–8 V, pulse duration 0.2 ms) were used to elicit all twitch contractions and trains of 0.2 ms pulses delivered at above fusion frequency for a sufficient time to reach a steady force plateau were used to elicit all tetanic contractions.

At the beginning of each experiment, muscles were equilibrated to 25°C and twitch contractions were performed at a range of stimulus amplitudes and the lowest voltage needed to produce peak twitch force determined. This voltage was used for all subsequent contractions. A series of twitch contractions were then performed at a range of muscle lengths and the muscle length that produced peak twitch force was defined as optimal length (*L*_0_) (Gordon et al., 1966). All subsequent contractions were performed at this length and total force was corrected for passive force at this length to determine active force. A tetanic contraction was then performed at optimal length to determine peak isometric force (*P*_0_).

### Temperature dependence of muscle contractile properties

Once *L*_0_ was determined and a baseline for *P*_0_ at 25°C was established, water bath temperature was adjusted to a random experimental temperature (15, 20, 25, 30 or 35°C, ±0.1°C). An additional isometric tetanic contraction was performed, and force–velocity properties were determined as detailed below at the selected temperature. The water bath was then adjusted back to 25°C for a final measurement of *P*_0_ that was used to compare initial and final force values to confirm muscle health. A thermocouple was placed in the muscle chamber to ensure temperature was consistent during each treatment. It should be noted that owing to slower force rise and relaxation times at lower temperatures, the stimulation frequency and train duration required to achieve fusion and a force plateau varied between 80 and 220 Hz and 300 and 500 ms. The minimum pulse frequency and train duration required to give a fused tetanic plateau for any given temperature were always used to preserve muscle viability and visual inspection was performed to ensure that fusion and a force plateau was always achieved.

For the determination of force–velocity properties, a series of approximately seven isotonic tetanic contractions, at forces ranging from 1% to 90% of *P*_0_, were used to characterize the force-velocity relationship of each muscle. The external resistive force provided by the servomotor was set to one of these relative force levels and the muscle stimulated to contract tetanically. Once the muscle exceeded the resistive force level, it shortened at some velocity under constant load. Peak muscle contractile velocity, and the force that was produced at that velocity, were then determined for each contraction and force–velocity curves constructed. Control tetanic contractions were done every 3–4 isotonic contractions at the test temperature, and at the end of the series at 25°C, to monitor muscle health. If peak isometric force fell below 80% *P*_0_ at the same temperature, data collection was halted. However, these muscles were typically very stable with sufficient contractions for force–velocity relationships at two to three temperatures that could be performed before any significant force loss.

#### Muscle morphology

Following the experiments, muscle belly length was measured to the nearest mm and weighed to the nearest 0.001 g for later calculations. Fiber length (*F*_L_) measurements were taken by placing muscles into a dissection tray, pinning at *L*_0_, and submerged into 10% formalin solution to fix for a minimum of 24 h. Using a digital camera mounted to a dissecting scope (Leica MZ9, Wetzlar, Germany), a series of photos were taken of each whole MTU, followed by a sagittal cross section through the aponeurosis that allowed the muscle fibers to be viewed. Using ImageJ software (NIH, USA), the length of three fiber lengths, distributed across the muscle belly, were measured and an average value taken. Owing to the lack of fiber length data on every individual, an average ratio of fiber length to muscle belly length was determined from 12 muscles and this ratio used to estimate *F*_L_ of muscles without direct measurements.

Physiological cross-sectional area (PCSA) was calculated using the equation:

(1) $$\mathrm{PCSA}=\frac{M_{\mathrm{muscle}}\times \cos\theta \;}{\rho \times F_{L}},$$

where ρ is muscle density (1.05 g cm^−2^; Gans and Bock, 1965) and θ is the pennation angle of the muscle measured relative to the line of action. Given the complex fiber structure of the gastrocnemius, it was difficult to determine θ consistently and measured θ was small (<20). Hence, pennation angle was subsequently removed from calculation of PCSA.

#### Data analysis

Maximal isometric stress was calculated by dividing *P*_0_ by PCSA. For each isotonic force–velocity contraction, peak absolute velocity was determined by differentiating muscle length change with respect to time and determining the peak velocity achieved during the contraction. Absolute velocity values were normalized to muscle fiber length to give a normalized velocity in fiber lengths per second (*L*_0_ s^−1^) to allow for comparison between individuals. Force (**F**) in each shortening contraction was taken at the time of peak velocity and then normalized to peak isometric force to give relative force (**F**/**F**_0_). Relative force was plotted against normalized velocity and a hyperbolic-linear curve was fit to the data using the methods following (Marsh and Bennett, 1986b):

(2) $$V=\frac{B(1-F/F_{0})}{\left(A+F/F_{0}\right)}+C(1-F/F_{0}),$$

where *V* is velocity in fiber lengths per second *L*_0_ s^−1^, **F**/**F**_0_ is force in Newtons as a fraction of maximum isometric force and *B*, and *C* are constants with dimensions of velocity, and *A* is dimensionless. Normalized maximum velocity (*V*_max_) was extrapolated from these force–velocity relationships. Muscle power was calculated as the product of absolute force and velocity at all points along the non-normalized fitted force–velocity curve, maximum power was determined and was divided by muscle mass to give maximal mass-specific muscle power (**W**_max_). *Q*_10_ values were then taken as the change in variables between 15 and 25°C or between 25 and 35°C.

#### Statistics

Statistical analyses were performed in R v. 4.0.3 (r-project.org) to test the effect of temperature on the contractile properties of the gastrocnemius in the western fence lizard. We tested for normality and homoscedasticity of each dataset using four diagnostic plots. Muscle contractile data were analyzed using a repeated measures linear mixed effects model (LMM) from the package lme4 (https://CRAN.R-project.org/package=lme4; Bates et al., 2015) to test for the effect of temperature on maximal tetanic stress, *V*_max_ and **W**_max_. The model for the analysis of *V*_max_ included individuals as a nested variable to account for individual variation, muscle mass was included as a covariate and temperature was the categorical independent variable. Multiple temperature treatments were done to the same muscle (until all temperatures were tested or the muscle health had deteriorated), and as such the use of a repeated measures analysis was required. The models for maximal isometric stress and **W**_max_ used the same parameters, however muscle mass was used in the calculation of each metric leaving no need for it as a covariate.

### Fixed-end contractions and potential for elastic strain energy storage

To determine the potential for elastic strain energy storage in the gastrocnemius muscle-tendon unit, an *in vitro* muscle preparation followed using the same initial methods as above, with the exception of temperature manipulation. Muscles were held at room temperature (∼22°C) during each trial. Animals were euthanized and the gastrocnemius MTU were dissected out. In this instance the whole distal tendon and connected plantar fascia were extracted. This allowed the inclusion of all possible elastic tissue and enabled us to tie onto the tissue without shortening length of the distal tendon. Following dissection, the muscle was placed into a shallow bath of oxygenated Ringer's solution and pinned at a length that removed the visible crimp in the tendons. Under a dissection scope, 4 fine sutures (6-0 silk) were placed at specific locations along the long axis of the muscle-tendon unit. Suture 1 was placed at the proximal end of the muscle near where it was to be clamped as a base marker to be used for total MTU length measurements. Suture 2 was placed on a muscle fascicle, as close as possible to proximal aponeurosis without contacting it. Suture 3 was placed at the distal end of the same fascicle as close as possible to the distal aponeurosis without contacting it. And suture 4 was placed near the tie-off point where the distal end of the tendon is connected to the servomotor (Fig. 1).

**Fig. 1.**
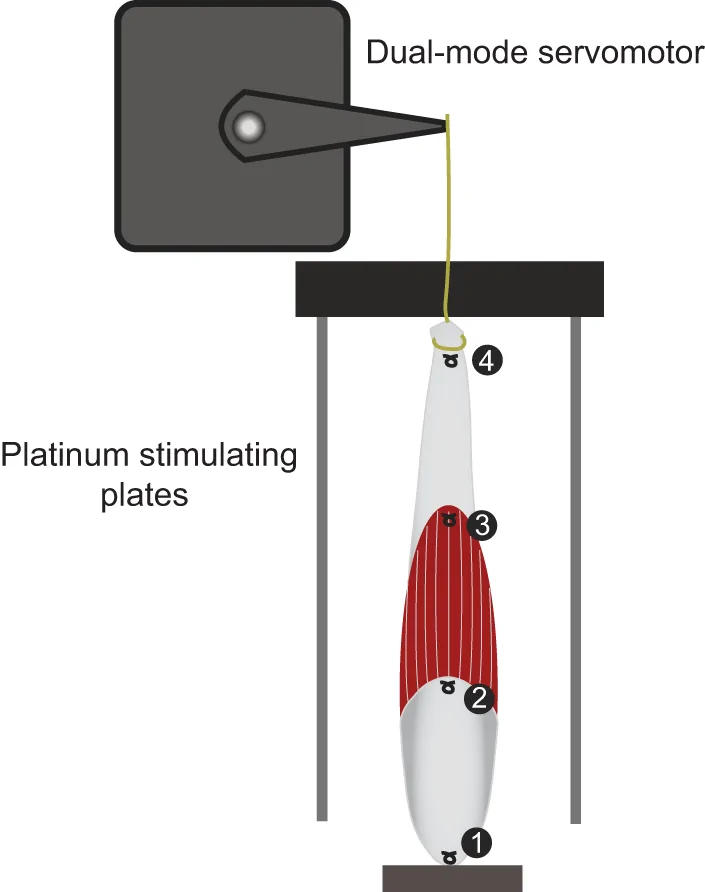
**A western fence lizard (*Sceloporus* *occidentalis*) gastrocnemius muscle–tendon unit in the experimental setup**. Sutures were placed along the long axis of the muscle–tendon unit (MTU) denote landmarks to be used for length estimation and displacement. Sutures 1–4 are muscle-tendon unit length. Sutures 1–3 are muscle belly length. Sutures 2–3 are approximations of muscle fiber length. Sutures 3–4 are tendon length.

After sutures were placed, the muscle was then attached by its distal end to a dual-mode servomotor (305C-LR dual-mode lever system, Aurora Scientific) via an inextensible Kevlar thread and its proximal end securely clamped into the stereotaxic frame. Stimulation voltage and optimal length (*L*_0_) were both set as above. A series of fixed-end tetanic contractions were carried out with train durations of 400 ms and stimulation frequency of 80 Hz, and the muscle was filmed. In fixed-end contractions the ends of the muscle tendon unit are fixed in place, and the muscle is stimulated to produce a maximal tetanic contraction. Muscle fibers shorten, stretching all SEEs, until a force equilibrium is reached. The percentage shortening that can be achieved by muscle fibers was taken as a measure of the fixed-end compliance of the muscle-tendon unit and provides a measure of the ability of a muscle to store elastic strain energy in its SEEs (Mendoza and Azizi, 2021; Holt and Mayfield, 2023). During fixed-end contractions, marker displacement was tracked, showing fiber shortening and tendon elongation. Maximal contractile force was then time synchronized with length recording from the video later analysis (described below). Peak tetanic isometric force was determined. Such a contraction is not representative of *in vivo* muscle function but rather demonstrates the likely upper limit of energy storage potential in this MTU.

#### Camera setup and video processing

Fixed-end contractions were filmed at 250 frames s^−1^ using two synchronized Edgertronic high speed cameras to obtain 3D video data (model SC2X, Sanstreak Corporation, San Jose, CA, USA). Cameras were placed at a 90 deg angle to one another with the muscle being the focal point, and eight calibration markers were placed on the muscle rig itself. Each contraction was filmed for 5 s to capture the onset of stimulation, contraction, and relaxation of the muscle. After each trial, videos were downloaded, trimmed and imported into MATLAB (MathWorks, Natick, MA, USA) for further analysis. DLTdv8a (Hedrick, 2008) software was used to digitize the four sutures placed along the MTUs to generate *X*, *Y* and *Z* coordinates for each suture through time. Data were then imported into Igor Pro 8 software (WaveMetrics) for further analysis.

#### Muscle morphology

Following the experiments, muscle belly length was measured to the nearest mm and weighed to the nearest 0.001 g. Muscle was then pinned at *L*_0_ and submerged into 10% formalin solution to fix for a minimum of 24 h. Following fixation, muscle was sagittally bisected to expose muscle fibers for measurement. Muscle fiber length, PCSA and stress were calculated using the methods described above.

### Data processing

The *X*, *Y* and *Z* data points for each suture were synchronized with relevant force data recorded in Igor. Force data were collected at 10,000 Hz, while the high-speed video was recorded at 250 Hz, requiring force data to be down sampled. The distances between sutures 1 and 4, 2 and 3, and 3 and 4 were measured in each frame. The distance between sutures 1 and 4 was the total MTU length. The distance between sutures 2 and 3 was the fiber length. And the distance between sutures 3 and 4 was the series elastic element length. Changes in whole MTU, fiber length and series elastic element length could then be determined over the time course of the contraction. In this fixed-end contraction, there should be no change in total MTU length. However, some small degree of compliance in the system is unavoidable and there was a very slight MTU shortening during each contraction. Fiber shortening was corrected for this to avoid overestimating muscle fiber shortening and work done on the series elastic element.

Fixed-end compliance and tendon strain were calculated as the percentage fiber shortening and series elastic element stretch, respectively. Work done by the muscle on the tendon was calculated by plotting muscle force against fiber length change during the fixed-end contraction and integrating the area under the curve. This work was divided by the mass of the muscle to get a measure of mass-specific elastic energy storage that could be compared with MTUs studied previously (Mendoza and Azizi, 2021).

## RESULTS

### Temperature dependence of muscle contractile properties

The maximum isometric stress of muscles at 25°C was 0.203 (N mm^−2^), slightly higher than 0.172 N mm^−2^ reported for the iliofibularis muscle in this species (Marsh and Bennett, 1986a). Temperature had relatively small effects on isometric stress over the 15–35°C temperature range examined here (Table 1). Average isometric stress decreased slightly from 0.182 N mm^−2^ at 15°C to 0.137 N mm^−2^ at 20°C, but then increased to 0.203 N mm^−2^ at 25°C. At higher temperatures, stress increased marginally to 0.229 N mm^−2^ at both 30 and 35°C. Though there was no statistically significant effect of temperature on stress (ANOVA, *F*=2.37, *P*=0.16), there was a general trend of increasing stress with increasing temperature (Table 1).

**Table 1.** Contractile properties of the gastrocnemius muscle of *Sceloporus occidentalis*

Temperature	*N*	Stress (N mm^−2^)	*V*_max_ (*L*_0_ s^−1^)	**F**_max_ (W kg^−1^)	Stimulation frequency (Hz)	Train duration (ms)
35	5	0.229±0.01	8.69±0.41	371±22.76	200–220	300
30	5	0.229±0.02	7.42±0.41	318±26.4	160–200	300
25	7	0.203±0.03	4.98±0.32	156±13.2	120–140	400
20	4	0.137±0.03	4.35±0.36	82.0±11.8	80	400
15	4	0.182±0.02	2.89±0.41	82.7±6.93	80	500

Data are means±s.e.m.

Maximum shortening velocity (*V*_max_) increased from 2.89 *L*_0_ s^−1^ at 15°C to 4.98 *L*_0_ s^−1^ at 25°C, and to 8.68 *L*_0_ s^−1^ at 35°C. *Q*_10_ values were 1.78 for 15–25°C and 1.74 for 25–35°C. Temperature had a significant effect on *V*_max_ (ANOVA, *F*=55.39, *P*<0.001), with *V*_max_ increasing with increasing temperatures (Fig. 2A). In line with the effects of temperature on force and velocity, maximum mass-specific muscle power increased from 82.7 W kg^−1^ at 15°C to 156 W kg^−1^ at 25°C, and to 371 W kg^−1^ at 35°C with a *Q*_10_ of 1.88 for 15–25°C and 2.38 and for 25–35°C. Temperature therefore also has a significant effect on maximum mass-specific muscle power (ANOVA, *F*=45.1, *P*<0.001; Figs 2B and 3).

**Fig. 2.**
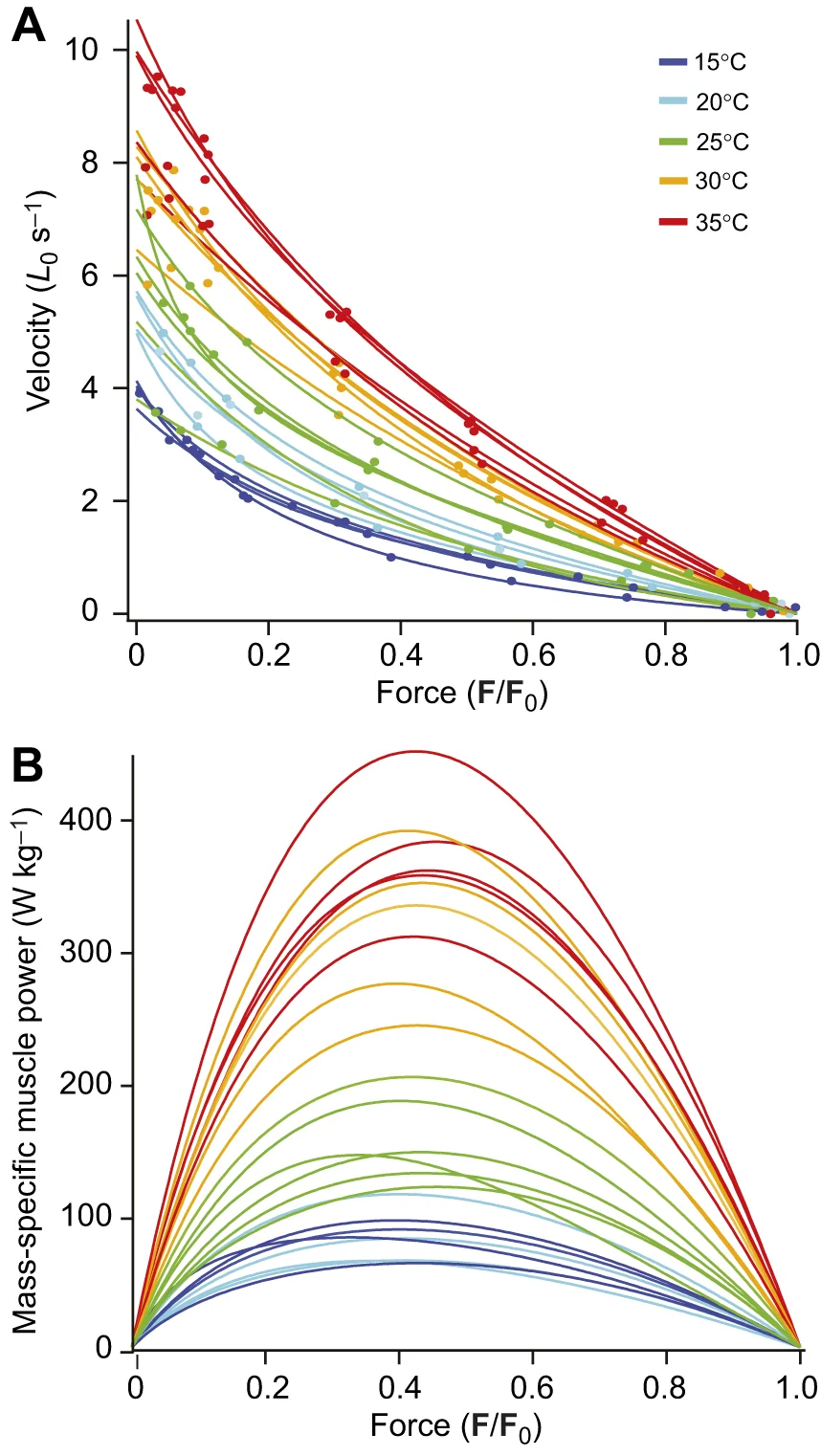
**Thermal dependence of muscle contractile properties.** (A) Force–velocity relationships for four representative individuals at each temperature and individual force–velocity curve fits. (B) Power curves of the same four representatives at each temperature calculated from the force–velocity fit.

**Fig. 3.**
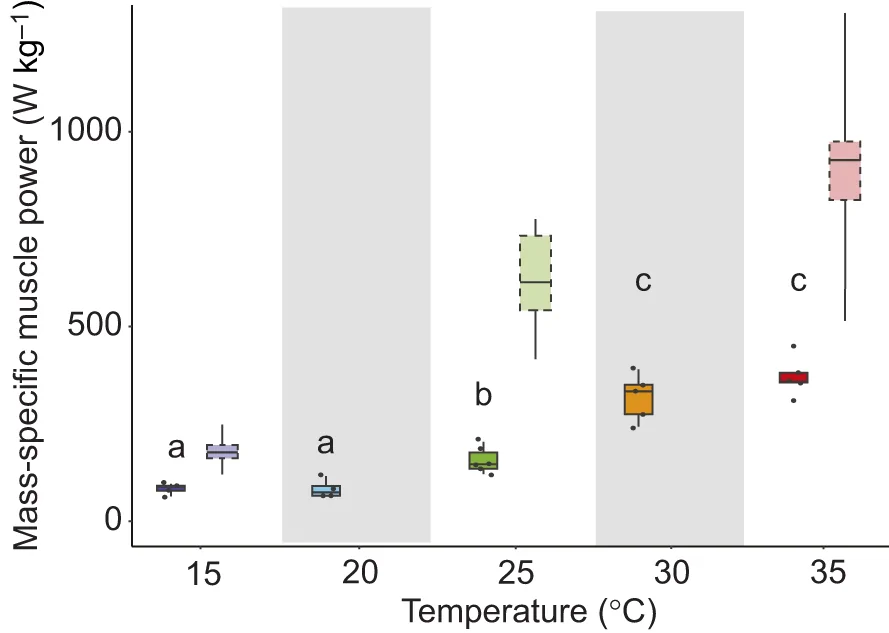
**Comparison of the mass-specific muscle power measured at each temperature with the maximum apparent instantaneous *in vivo* muscle power at the ankle during sprinting.** Data from this study (dark boxes; left of each temperature column) and from our companion study (light boxes; right of each temperature column for 15, 25 and 35°C) (Balaban et al., 2026). Boxes show median, minimum, 25th percentile, 75th percentile and maximum datapoints; raw data points from this study are overlaid. Average measured muscle power increases as temperatures increase, different letters above treatments indicate statistically distinguishable groupings based on Tukey's HSD *post hoc* test. *N* values for each temperature for the data presented in this study are as given in Table 1 (left of columns). Only summary data from Balaban et al. (2026) are presented (right of columns).

### Fixed-end contractions and potential for elastic strain energy storage

Fixed-end compliance was measured during tetanic fixed-end contractions at 25°C in which muscles produced 1.53±0.14 N force and had a stress of 0.18±0.015 N mm^−2^ (*n*=5). Muscle fibers shortened by 0.83±0.11 mm giving fixed-end compliance values of 16.7±2.17% (*n*=5). Tendon stretched by 0.89±0.08 mm resulting in a tendon strain of 11.8±1.69% (*n*=5). The work done by muscle on the tendon, the integral of muscle force and length change (Fig. 4), was 0.87±0.18 J. When normalized to muscle mass this gave a muscle mass-specific energy storage of 17.3±2.52 J kg^−1^ (*n*=5) (Fig. 5).

**Fig. 4.**
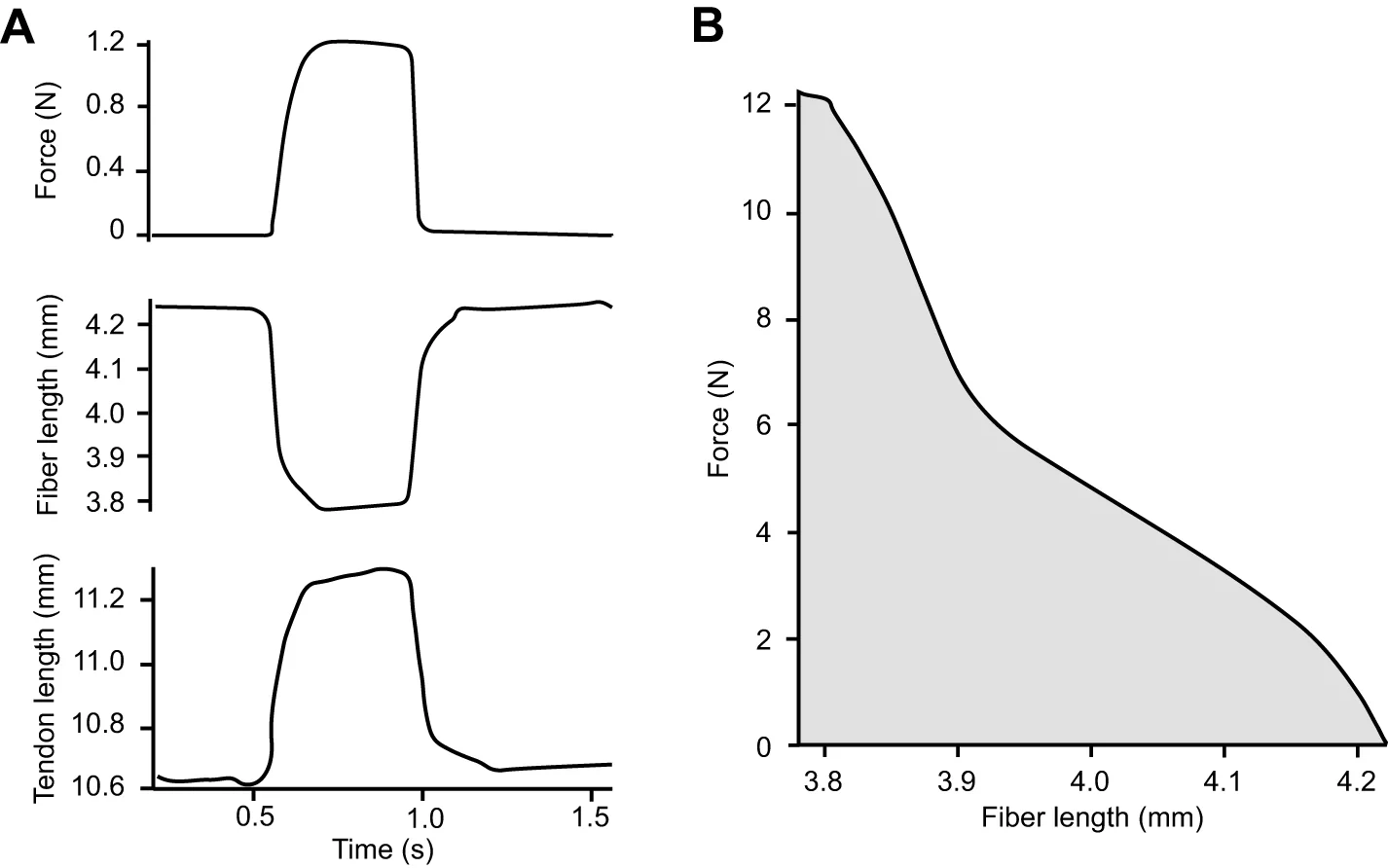
**Fixed end compliance and elastic strain energy storage.** (A) Representative time series trace for a single fixed-end contraction showing active muscle force generation (top), muscle fiber shortening (middle) and tendon stretch (bottom). (B) Force and fiber length data from A plotted against one another with the area under this curve highlighted to show the work done by muscle fibers on series elastic elements.

**Fig. 5.**
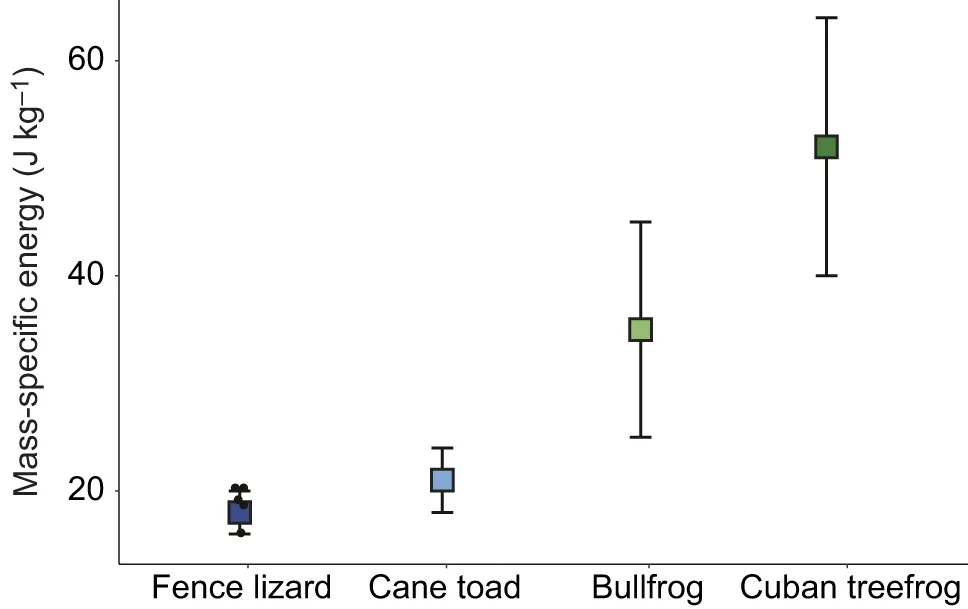
**Comparison of mass-specific elastic strain energy storage in the western fence lizard demonstrated in this study (*N*=5) with three anuran species known to utilize elastic strain energy in power amplification (adapted from Mendoza and Azizi, 2021).** Data are means±s.e.m. Raw data points from this study are overlaid.

## DISCUSSION

Here, we examined the effect of temperature on contractile performance of gastrocnemius muscle, and the potential for this MTU to store elastic strain energy in its SEEs, in the western fence lizard (*Sceloporus occidentalis*) to better understand the previously reported mismatch between muscle performance, and joint and organismal performance across a thermal range (Marsh and Bennett, 1986a,b; Balaban et al., 2026). Sprint performance and ankle joint power, and the thermal robustness of these properties, have been shown to exceed that predicted by muscle alone, implicating elastic strain energy storage and power amplification. However, the power output of this muscle, its temperature sensitivity, and potential for SEEs to store elastic strain energy were previously unknown. The environmental temperatures experienced by *S. occidentalis* range from ∼15 to 40°C, with lizards often shuttling between different microhabitats to maintain a preferred body temperature of ∼33°C (Burgos, 2018). Hence, the 15–35°C range examined here represents a physiologically relevant range of temperatures and includes preferred body temperature and thus, our findings have implications for the link between physiological and morphological traits and locomotor performance under ecologically relevant conditions.

### Temperature dependence of muscle contractile properties

Here, we observed a non-significant trend of increasing peak isometric stress with increasing temperature. This is largely consistent with a slight increase in peak isometric stress of the iliofibularis with increasing temperature in this species (Marsh and Bennett, 1986b) and across both ectotherms (Marsh and Bennett, 1986a,b; Peplowski and Marsh, 1997) and endotherms (Rall and Woledge, 1990; Ranatunga, 2018) more broadly. This decline in force is attributed to a decrease in the force per attached crossbridge at low temperatures (Huxley, 1957; Ford et al., 1977; Johnston and Gleeson, 1984; Goldman et al., 1987; Coupland et al., 2001; Linari et al., 2005; Piazzesi et al., 2003; Herrel et al., 2007). Any variation in temperature sensitivity appears to be dependent on the thermal life history of the taxa, with icefish that are active at −1°C maintaining tetanic force production over the 0–10°C range (Johnston and Altringham, 1985), whereas desert iguanas which are active at 35–42°C show a decline in force below 25°C (Johnston and Gleeson, 1984). However, we also note a strong temperature dependence of stimulation fusion frequency (Table 1) and a large effect of stimulation frequency on peak force (Fig. S1), which may account for some of apparent variation in thermal sensitivity as the treatment of stimulation frequency varies widely across studies (Renaud and Stevens, 1984; Herrel et al., 2007; Balaban and Azizi, 2017; Olberding and Deban, 2017; Moran et al., 2019; Niu et al., 2021; Rummel et al., 2021; Mendoza et al., 2023a). The maintenance of isometric stress across temperatures reported here, and demonstrated previously, is potentially advantageous as it could allow for sufficient force production to stretch elastic structures over a large temperature range (Mendoza and Azizi, 2021).

In contrast to the relative thermal insensitivity of peak isometric stress, we demonstrate a strong dependence of *V*_max_ on temperature with a *Q*_10_ value of ∼1.76 over the temperature range examined here. This is consistent with the increase in *V*_max_ with temperature documented across vertebrates (Hill, 1938; Ranatunga, 1982, 1984; Putnam and Bennett, 1982; Johnston and Gleeson, 1984; Marsh and Bennett, 1986b; Bennett, 1990; Rome and Sosnicki, 1990; Swoap et al., 1993; Coughlin et al., 1996; James, 2013; Balaban and Azizi, 2017) and is comparable to the *Q*_10_ of ∼1.7 reported in the iliofibularis muscle of this species (Marsh and Bennett, 1986b). As with force, variation in *V*_max_ also appears to be correlated to the thermal environment experienced by each species (Putnam and Bennett, 1982). Functionally, operating across temperatures equally is difficult, if not impossible, for species adapted to specific thermal ranges, as muscle tissue is more temperature sensitive at the relatively lower temperatures experienced by animals in their respective environments (Johnston and Gleeson, 1984; Ranatunga, 1984; Bennett, 1990; Rall and Woledge, 1990). For example, species such as the desert iguana that are specialized for surviving in high thermal ranges, experience a significant decline in muscle performance at low temperatures (i.e. 5–15°C), with a *Q*_10_ of contractile velocity as high as 4 (Johnston and Gleeson, 1984). This value decreases to 2.5 between 15 and 25°C and further to 1.5 between 25 and 35°C (Johnston and Gleeson, 1984). This shift in thermal dependence typically occurs close to the muscle temperatures encountered by the animal in its environment (James, 2013), as is what is apparent with the western fence lizard in both its gastrocnemius and iliofibularis muscles. The western fence lizard has a preferred body temperature of ∼33°C (Burgos, 2018), which is consistent with our highest recorded *V*_max_ at 35°C (Fig. 2). However, we cannot exclude the potential for faster velocities at higher *in vitro* temperatures.

Maximum muscle power output, the product of force and velocity, showed a similar trend to *V*_max_, increasing with increasing temperature. The *Q*_10_ values reported here were 1.88 between 15 and 25°C, and 2.38 between 25 and 35°C. These values are relatively low, but broadly within the range of previously measured values (Bennett, 1984). However, trends in *Q*_10_ appear to show a different pattern with increasing temperature compared with other ectothermic species, increasing with increasing temperature whereas other studies have shown them to decrease. *Q*_10_ values of 4.25 at 5–15°C and 2.15 at 15–25°C have been reported for *Bufo americanus* (Renaud and Stevens, 1984), and *Q*_10_ has been reported to decline from ∼3 at 10°C to ∼1.5 at 25°C across a range of ectothermic species (Bennett, 1984). The mechanism for the low temperature sensitivity of muscle power at low temperatures reported here is unclear, and may be due to fundamental differences between muscles and/or differences in experimental protocols such as the tuning of optimal stimulation frequency. If this low temperature sensitivity arises from differences between muscles, it may contribute to the low thermal sensitivity of organismal performance observed in this species.

One goal of this study was to provide empirical support for the prediction that muscle power is insufficient to explain joint and organismal performance across a range of temperatures (Marsh and Bennett, 1986a; Balaban et al., 2026). Here, we find that the mass-specific muscle power of the gastrocnemius was ∼80, 155 and 370 W kg^−1^ at 15, 25 and 35°C, respectively. In the accompanying paper (Balaban et al., 2026) we showed that maximum instantaneous muscle power at the ankle was ∼180, 620 and 880 W kg^−1^ at 15, 25 and 35°C, respectively. Hence, maximum instantaneous muscle power observed at the joint dramatically exceeds the peak power available from muscle (Fig. 3); muscle power is amplified 2.2 times at 15°C, 4.1 times at 25°C, and 2.4 times at 35°C.

### Potential for elastic strain energy storage

In order for muscle power, or kinetic energy (Labonte and Holt, 2024), to be amplified, elastic strain energy must be stored in series elastic elements. Here, we performed fixed-end contractions in the gastrocnemius MTU and tracked muscle fiber length and series elastic element (i.e. tendon and associated distal plantar fascia) stretch via the filming of sutures implanted at various points along the MTU (Fig. 1) to determine the potential for this MTU to store elastic strain energy in the series elastic elements. This measurement is technically challenging because of the small size of the MTU (muscle belly ∼10 mm and series elastic elements ∼9 mm in length) and the complex morphology of this bipennate muscle. However, use of sutures rather than the larger sonomicrometry crystals (Roberts and Dick, 2023; Mayfield and Holt, 2025) enabled tracking of muscle fiber and series elastic element length, and there was good agreement between muscle force, muscle fiber shortening and tendon stretch (Fig. 4) so giving us confidence in our measurements of these small length changes.

Using these methods, we determined that the gastrocnemius MTU of this species has a fixed end compliance of ∼17% that the muscle can do ∼17 J kg^−1^ of work on the tendon that can be stored as elastic strain energy during maximal tetanic fixed end contractions. Fixed-end compliance varies widely across taxa and muscles owing to the relative length of the muscle fibers and series elastic elements, and the properties of these tissues (Zajac, 1989; Pollock and Shadwick, 1994; Mendoza and Azizi, 2021; Holt and Mayfield, 2023). Values reported for systems with known series elasticity range from 10% in mouse tibialis anterior muscle (Moo et al., 2020) to ∼45% in the Cuban tree frog (Mendoza and Azizi, 2021). So, while the ∼17% reported here for the western fence lizard is not as extreme as the exceptional power amplification system of the Cuban tree frog, it is comparable to the guinea fowl lateral gastrocnemius values (*Numida meleagris*, 13%; Buchanan and Marsh, 2001) and the leopard frog gastrocnemius (*Rana pipens*, ∼21%; Trestik and Lieber, 1993), both of which are known to amplify or enhance power during jumping (Henry et al., 2005).

In order to understand the significance of the ability to the western fence lizard to store ∼17 J kg^−1^ of elastic strain energy per unit muscle mass, we compared this with values reported in the cane toads, bullfrogs and Cuban tree frog plantaris muscles as power amplification has been studied most extensively in anuran jumping and has been demonstrated in all these systems power (Peplowski and Marsh, 1997; Astley and Roberts, 2014; Olberding and Deban, 2017; Mendoza and Azizi, 2021). We found that the western fence lizard is able to store muscle work as elastic strain energy on a comparable scale to these jumping species, being similar in storage capability to cane toads (Fig. 5).

### Power amplification

Here, we demonstrate insufficient gastrocnemius muscle power to provide the maximum instantaneous power observed at the ankle joint during sprinting across temperatures, and the capacity for elastic strain energy storage in the series elastic elements of this muscle. These findings provide strong support for power amplification during sprinting in the western fence lizard. This power, or kinetic energy, amplification (Labonte and Holt, 2024) may both enhance locomotor performance in general (Peplowski and Marsh, 1997; Aerts, 1998; Azizi and Roberts, 2009; Longo et al., 2019) and may increase its thermal robustness (Deban and Lappin, 2011; Anderson and Deban, 2010; Deban and Scales, 2016). Power amplification enhances organismal performance by using elastic structures to temporally decouple muscle work from movement (Roberts and Azizi, 2011). Historically, this has been assumed to overcome the force–velocity constraints of muscle (Roberts and Marsh, 2003). However, more recent work has suggested that, beyond the limitations of the force–velocity relationship, small animals are likely to be limited in the work they can deliver by the speed at which they are able to shorten, or their kinetic energy capacity (Labonte, 2023; Labonte and Holt, 2024). In either case, the rapid return of stored elastic strain energy by series elastic element recoil is thought to allow muscle to deliver more work (Roberts and Marsh, 2003; Labonte and Holt, 2024).

Power amplification has been most clearly demonstrated in ‘one-shot’ activities, such as jumping and predatory strikes. In these cases, muscle does work prior to movement, and a latching system prevents this muscle work from causing joint rotation and movement, directing it instead into elastic strain energy (Peplowski and Marsh, 1997; Aerts, 1998; Galantis and Woledge, 2003; Deban et al., 2007; Van Wassenbergh et al., 2008; Patek et al., 2011; Astley and Roberts, 2012; Ilton et al., 2018). In cyclical movements, such as running, there is less opportunity for this slow loading of elastic strain energy prior to movement and in steady state running series elastic elements are thought to provide benefits by reducing the need for metabolically costly muscle work (Roberts et al., 1997; Roberts and Azizi, 2011; Holt et al., 2014). However, power enhancement or amplification (Richards and Sawicki, 2012) has been demonstrated at push-off during human walking (Hof et al., 1983; Fukunaga et al., 2001; Lichtwark and Wilson, 2006), at the onset of swing in galloping horses (Wilson et al., 2003), and in the second half of stance in accelerating turkeys (Roberts and Scales, 2002) and humans (Lai et al., 2016; Miller et al., 2012; Lim et al., 2022). Also, simulations of human running suggest that muscle fibers shorten early in stance while the whole MTU is stretching, prolonging the time available for muscle work and allowing elastic strain energy storage (Lai et al., 2016).

Here, we show up to fourfold amplification of muscle power during a high work demand cyclical activity at the ankle joint of the small-bodied western fence lizard at 25°C. This appears to be a greater degree of power amplification than has been observed in other cyclical activities (Fukunaga et al., 2001; Roberts and Scales, 2002; Lichtwark and Wilson, 2006; Lai et al., 2016). However, it is thought that the benefits of power or kinetic energy amplification are likely restricted to small body sizes during jumping (Sutton et al., 2019; Labonte, 2023; Labonte and Holt, 2024), consistent with this apparent greater degree of power amplification during running.

Although power amplification probably enhances locomotor performance in the western fence lizard, it may also enhance its thermal robustness. Muscle contractile velocity, and therefore muscle power, is severely reduced at low temperatures (Hill, 1938; Ranatunga, 1982, 1984; Johnston and Gleeson, 1984; Rome and Sosnicki, 1990; Coughlin et al., 1996; James, 2013) making elastic recoil an effective means of retaining organismal performance at low temperatures in ectotherms (Deban and Lappin, 2011; Anderson and Deban, 2010; Deban and Scales, 2016). For example, chameleons can capture prey at near-freezing temperatures using elastically powered ballistic tongue projection (Anderson and Deban, 2010). The high degree of power amplification observed here in the western fence lizard at 25°C may allow for its previously observed thermal robustness of organismal performance in which there is little decline in sprint speed as lizards are cooled from 35 to 25°C (Marsh and Bennett, 1986a; Balaban et al., 2026). However, the lower degree of power amplification combined with reduced muscle power at 15°C makes it unlikely that performance can be maintained at lower temperatures. The situation at 35°C is a little harder to interpret. While there is still significant power amplification, there is less than at 25°C, which probably adds to the similarity of sprint performance at these temperatures. It is not clear why power amplification is reduced at 35 compared with 25°C. Given the difficulty in controlling the flow of energy in cyclical, compared with ‘one-shot’ activities, it may simply be that a limb coordination pattern that enables such a high degree of power amplification is not possible at 35°C. However, the accompanying study (Balaban et al., 2026) suggests that, in addition to reduced power amplification, muscle activation around the hip is also reduced at this high temperature. This suggests that there may be some other limit to acceleration and sprint performance and higher levels of sprint performance may not be achieved irrespective of greater muscle power or power amplification.

### Conclusions

Here, we show a relatively low thermal sensitivity of gastrocnemius muscle power of the western fence lizard over a physiologically relevant range of temperatures. Comparison of these muscle powers with maximal apparent instantaneous *in vivo* muscle power at the ankle joint, around which this muscle acts, from the previous study, suggests a high degree of power amplification at all temperatures, but particularly at 25°C. We also show that this MTU has a fixed-end compliance comparable with MTUs known to utilize elastic strain energy storage, and that the series elastic elements of this MTU can store an amount of elastic strain energy comparable to that in jumping anuran species known to amplify muscle power. This provides support for the amplification of muscle power by series elastic elements increasing the thermal robustness of sprint performance between 25 and 35°C. However, the lower power at high compared with intermediate temperatures observed here, and the apparent reduction in proximal muscle activation during sprinting at this high temperature observed in the previous study suggest that some other, currently unknown, limits to sprint performance, at high temperature may also contribute to this high thermal robustness.

## Supplementary Material

10.1242/jexbio.252629_sup1 Supplementary information
