# Supplementary material for: Muscle power amplification may enhance sprint performance and broaden the thermal performance of the lizard Sceloporus occidentalis. II: Muscle power and series elastic element properties
Source: J Exp Biol. 2026 Oct 23;229(20):jeb252629. doi: 10.1242/jeb.252629 (PMC13723398; doi:10.1242/jeb.252629)
Supplement: Supplementary file 1 [file jexbio-229-252629-s1.pdf]

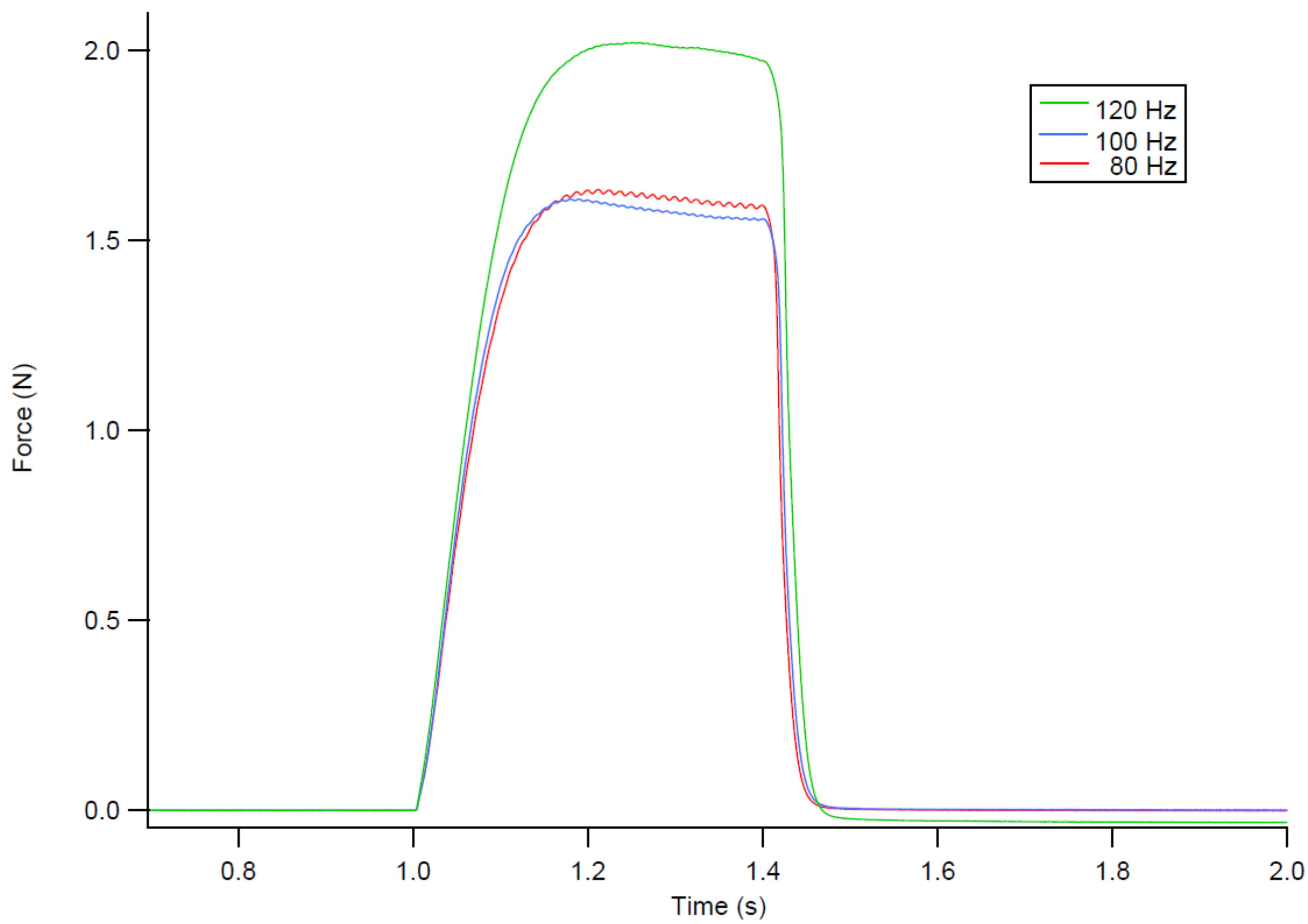

Fig. S1. Time series force data for 3 stimulation frequencies at 25 °C illustrating the large increase in force between 100 and 120 Hz despite the very high degree of fusion observed at 100 Hz. Force did not continue to rise at stimulation frequencies greater than 120 Hz and often declined during the course of the contraction, presumably due to fatigue.

Journal of Experimental Biology • Supplementary information
